# Penile Glans Necrosis Associated With Antiphospholipid Syndrome: A Rare Complication

**DOI:** 10.7759/cureus.39999

**Published:** 2023-06-05

**Authors:** Minh H Truong, Trung Q Ngo, Thang D Vu

**Affiliations:** 1 Department of Urology and Renal Transplantation, People's Hospital 115, Ho Chi Minh, VNM; 2 Department of Intensive Care Unit and Poison Control, People’s Hospital 115, Ho Chi Minh, VNM

**Keywords:** ischemia of the penis, catastrophic antiphospholipid syndrome (caps), penile reconstructive surgery, antiphospholipid syndrome, penile glans necrosis

## Abstract

Penile glans necrosis is a rare clinical condition caused by trauma, diabetes mellitus, adverse effect of vasoconstrictive solutions, and circumcision. Antiphospholipid syndrome (APS) is categorized as an autoimmune disease with the presence of antiphospholipid antibodies that results in an increased risk of vascular thrombosis and obstetrical complications. In this article, we report a rare case of a 20-year-old boy with penile glans necrosis due to penile vascular thrombosis following catastrophic antiphospholipid syndrome (CAPS) which we successfully treated at People’s Hospital 115.

## Introduction

The ischemia or necrosis of the glans penis is an uncommon condition, and the etiologies of which could be traumatic, vasculogenic, complications of vasoconstrictive agents, and surgical procedures such as circumcision, prostatic artery embolization, or penile cosmetic procedures [1-4]. This disease could be diagnosed by the glans appearance. Antiphospholipid syndrome (APS) is a thrombo-inflammatory disease characterized by the existence of autoantibodies including anticardiolipin, anti-β2-glycoprotein 1, and lupus anticoagulant that is directed against phospholipid-binding plasma proteins. Consequently, it manifests recurrent thrombosis in the veins, arteries, microvasculature, and pregnancy complications. The APS is estimated to affect 40 to 50 cases per 100 000, with a yearly prevalence of 1 to 2 per 100 000. In addition, patients with the diagnosis of APS are comparatively younger individuals, and only 12.7% of patients had it diagnosed after the age of 50 [5]. In the case of catastrophic antiphospholipid syndrome (CAPS), its manifestations become rapid and severe with thrombosis in various vascular beds, resulting in multiorgan failure and a high fatality rate [6].

In this article, we present a rare case of penile glans necrosis following CAPS, and we successfully performed the penile reconstructive surgery with two stages. After the surgeries, the patient feels quite satisfied with urinary function as well as erectile function.

## Case presentation

A 20-year-old male presented to our hospital with severe hematuria and dull pain in his left iliac fossa. The initial onset of the disease was 10 days prior to hospital admission, and he had a sore throat and a dry cough. He received outpatient treatment and the condition was relieved. Six days later, he suddenly had severe hematuria. The urine was heavily blood-stained and had clots, in tandem with progressive acute kidney injury, coagulation disorders, and severe anemia (Table 1). The patient was admitted and monitored in the ICU.

**Table 1:** Laboratory values before admission to the ICU WBC: white blood cell, INR: international normalized ratio, aPTT: activated partial thromboplastin time, CRP: c-reactive protein, ASLO: antistreptolysin O

Lab	Value	Reference range
WBC count	27	4.0-10.0 K/µL
Absolute neutrophile	21.74	2.5-7.0 K/µL
Hemoglobin	11.6 → 8,2 → 4,8	12.2-15.4 g/dL
Platelet count	131 → 61	150-400 K/µL
INR	Max	1.14-1.27 s
aPTT	Max	-
D-dimer	>20	<0.25 µg/mL
Fibrinogen	1.13	2-4 g/L
Serum urea	24.50	1.7-8.3 mmol/L
Serum creatinine	262 → 429 → 775	62-106 umol/L
Serum albumin	26	34-48 g/L
CRP	88.45	<6 mg/L
ASLO	1521.55	0-200 UI/ml
Coombs tests	Coomb D, I (++)	Negative
24h urine protein	7.9	<0.1 g/24h

**Figure 1:**
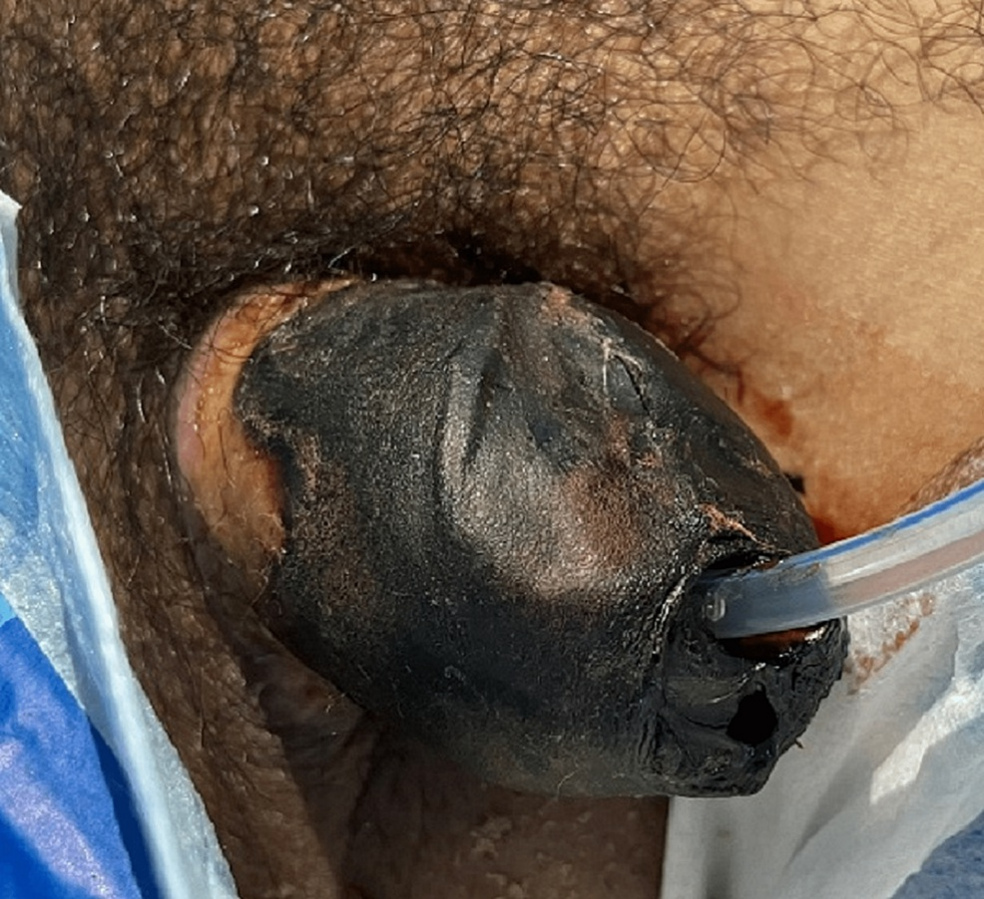
The necrosis of the glans penis on the 14th day of the disease

On the 14th day of illness, he had the symptoms of acute respiratory failure, and the thrombus occurred at multiple vascular sites. Vascular Doppler ultrasound recorded the thrombosis in the bilateral cephalic veins, the bilateral great saphenous and small saphenous veins, and the thrombosis in the testicular and penile arteries. Dry gangrene appeared sporadically on the extremities, scrotum, and penis (Figure 1). The blood tests gave a positive for antiphospholipid IgM that oriented the diagnosis as APS (we also repeated the serum antibodies test after that 12 weeks to confirm the diagnosis with the following results: anti-cardiolipin IgG (+) (>99 percentile) and anti-phospholipid IgG (+)). The patient received intensive care with a high-flow nasal cannula, plasma exchange, continuous renal replacement therapy, heparin infusion, and corticosteroid therapy. After that, the patient’s condition progressed better. We also performed a suprapubic trocar cystostomy and took care of necrotic wounds daily.

**Figure 2:**
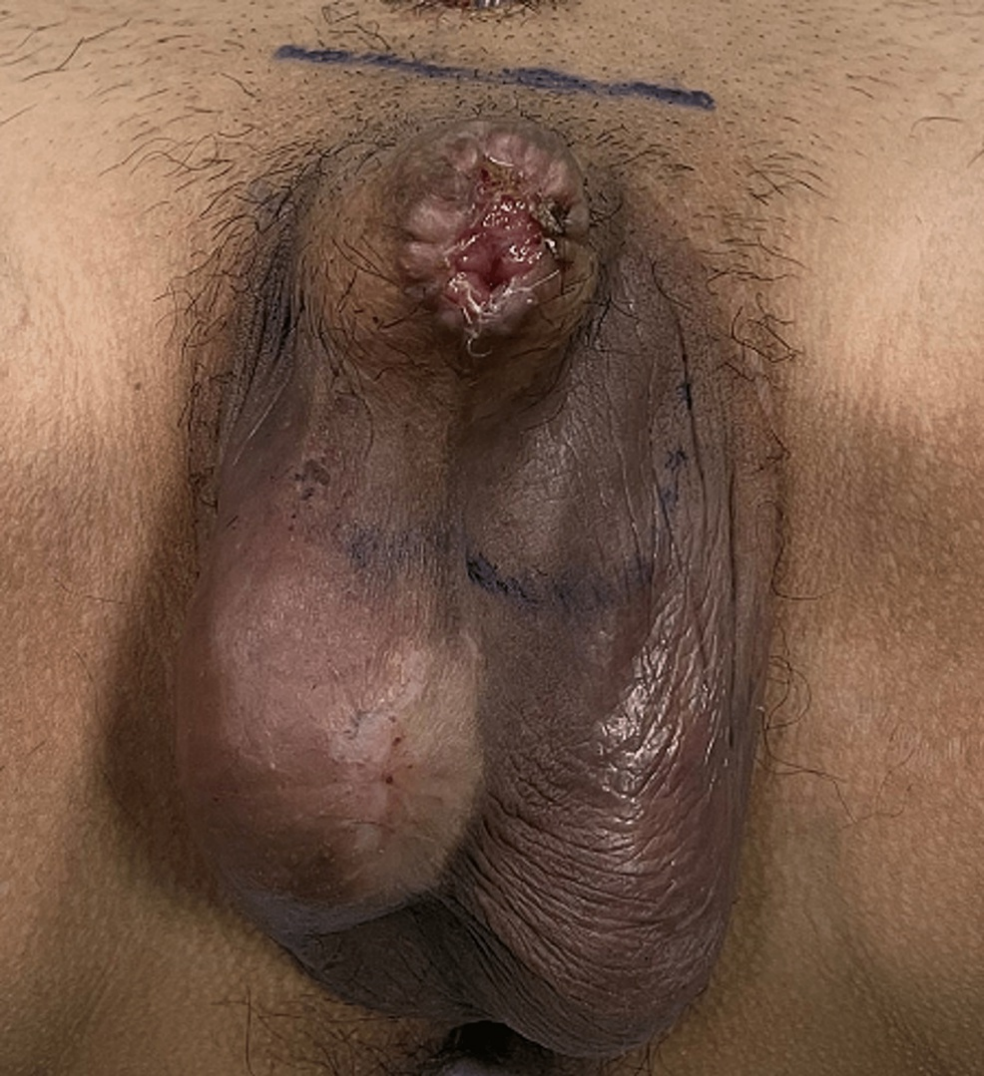
The penis after the first reconstructive surgery with urethral reconstruction and burying the penis into the penile healthy skin left and the scrotum

**Figure 3:**
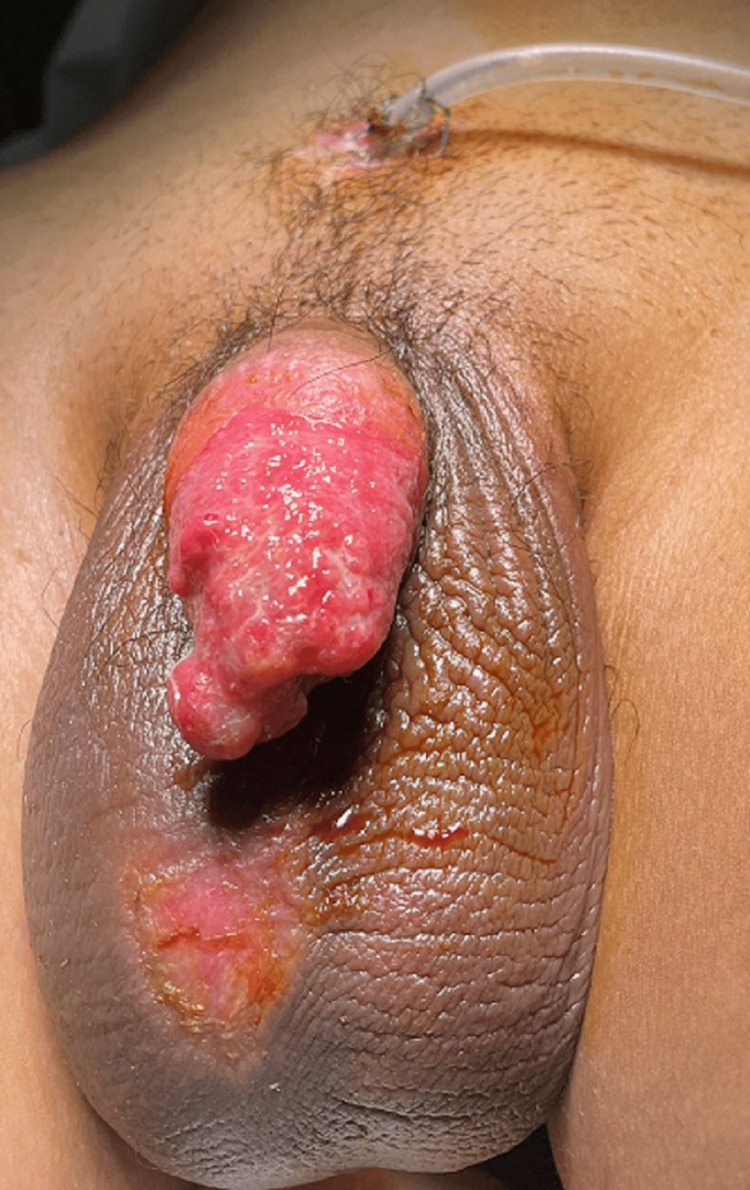
The penis after eliminating the glans necrosis

The patient’s condition stabilized after a month, recovering glomerular filtration rate and under control coagulation disorders. We debrided penile glans necrotic tissues. Due to dry gangrene of the embolism, the patient lost the whole glans penis and the urethra sloughed partially (Figure 2). Two weeks later, the patient underwent surgery to reconstruct the urethra and bury the penis into the remaining penile skin flap and scrotal skin. There was good postoperative progress, and after removing the Foley catheter and cystostomy tube, he could urinate easily and was sent home seven days later (Figure 3).

**Figure 4:**
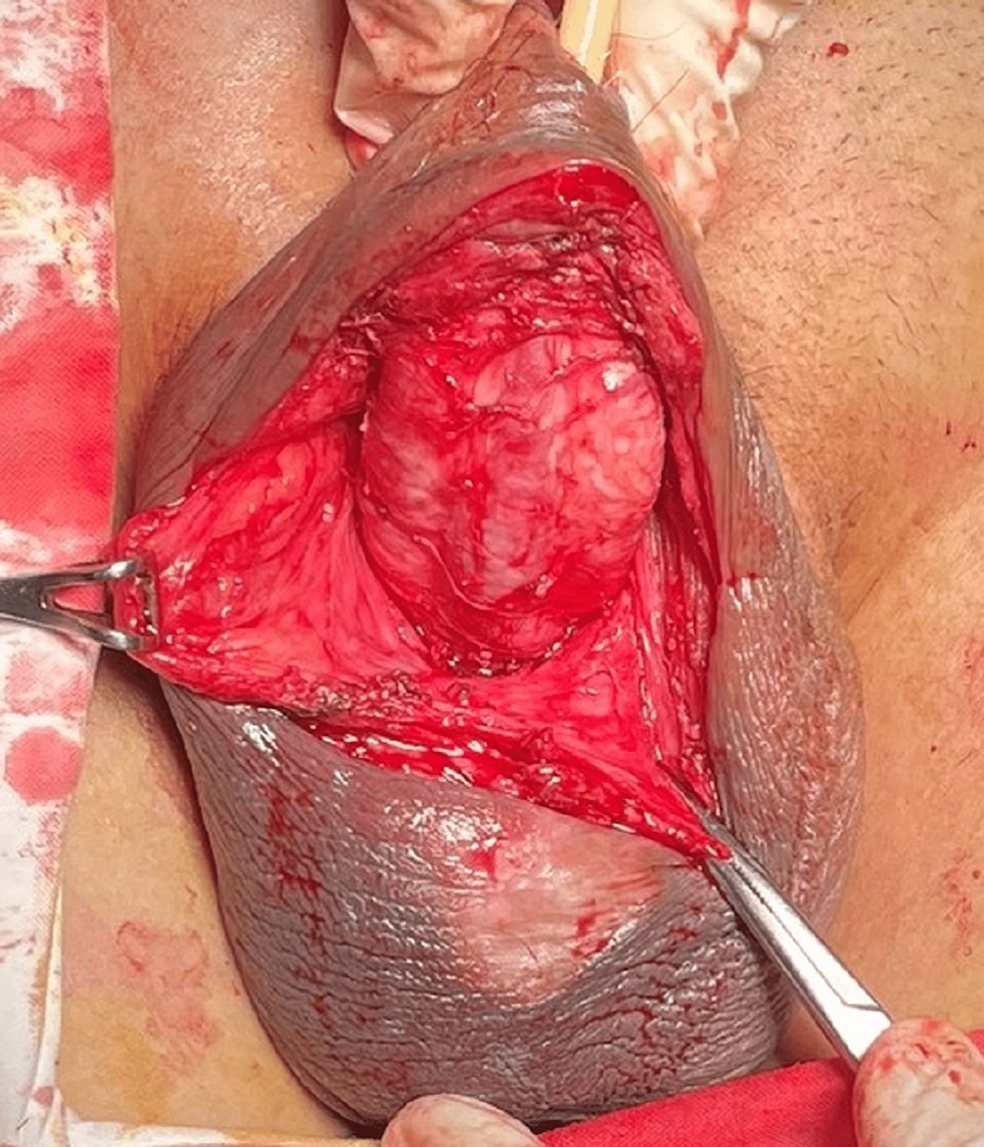
Modified Lipszyc technique for the buried penis

**Figure 5:**
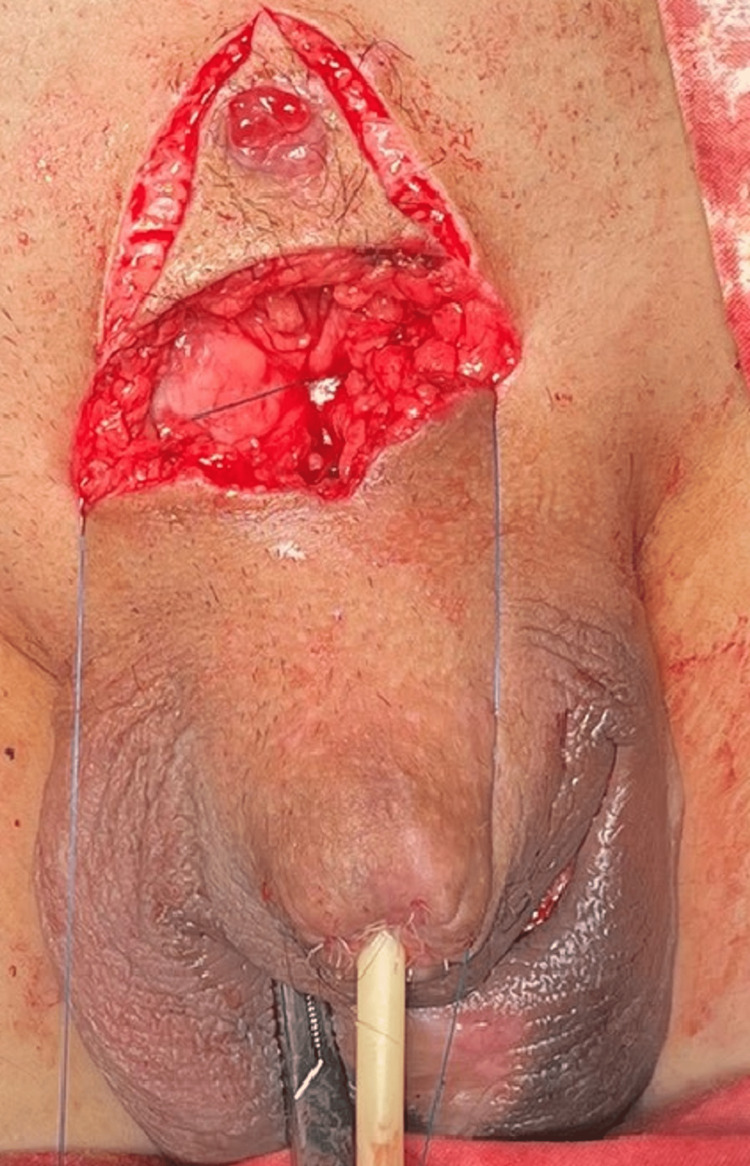
Pubic lipectomy and cutting of the suspensory ligament of the penis

**Figure 6:**
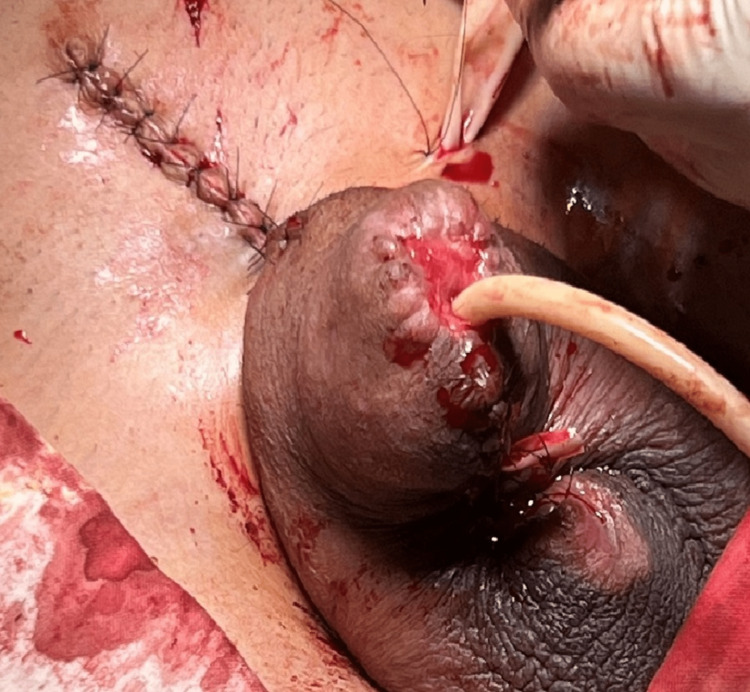
The penis after the second stage of reconstructive surgery

**Figure 7:**
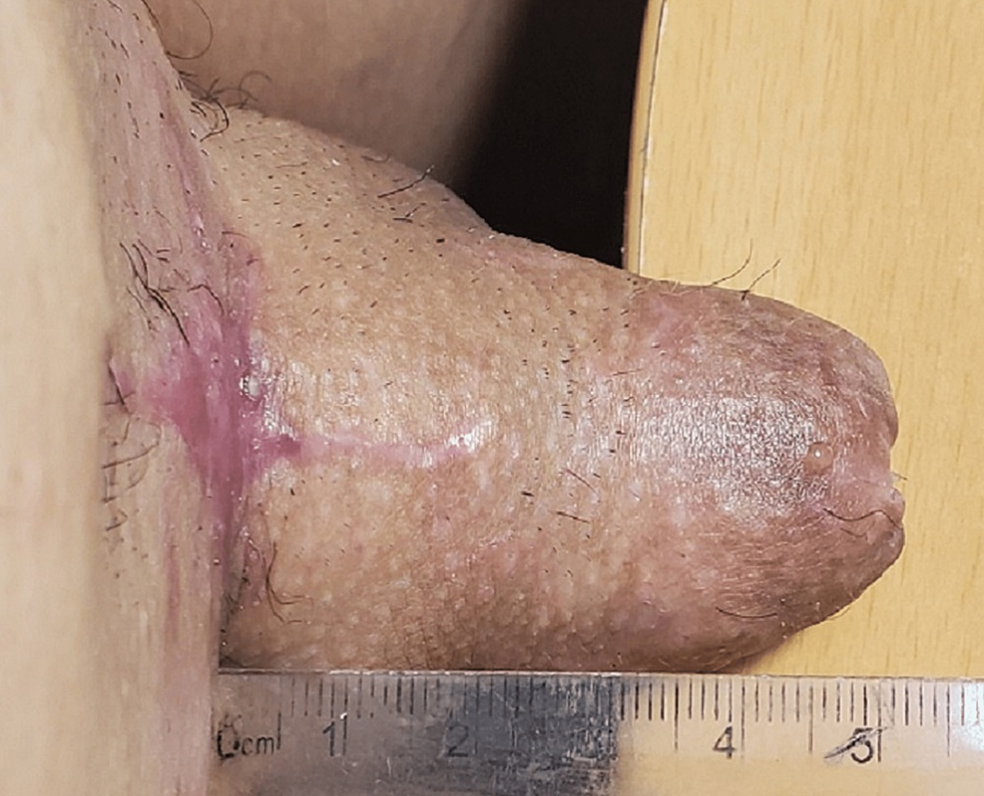
The penis after completely reconstructive procedures in the flaccid state

**Figure 8:**
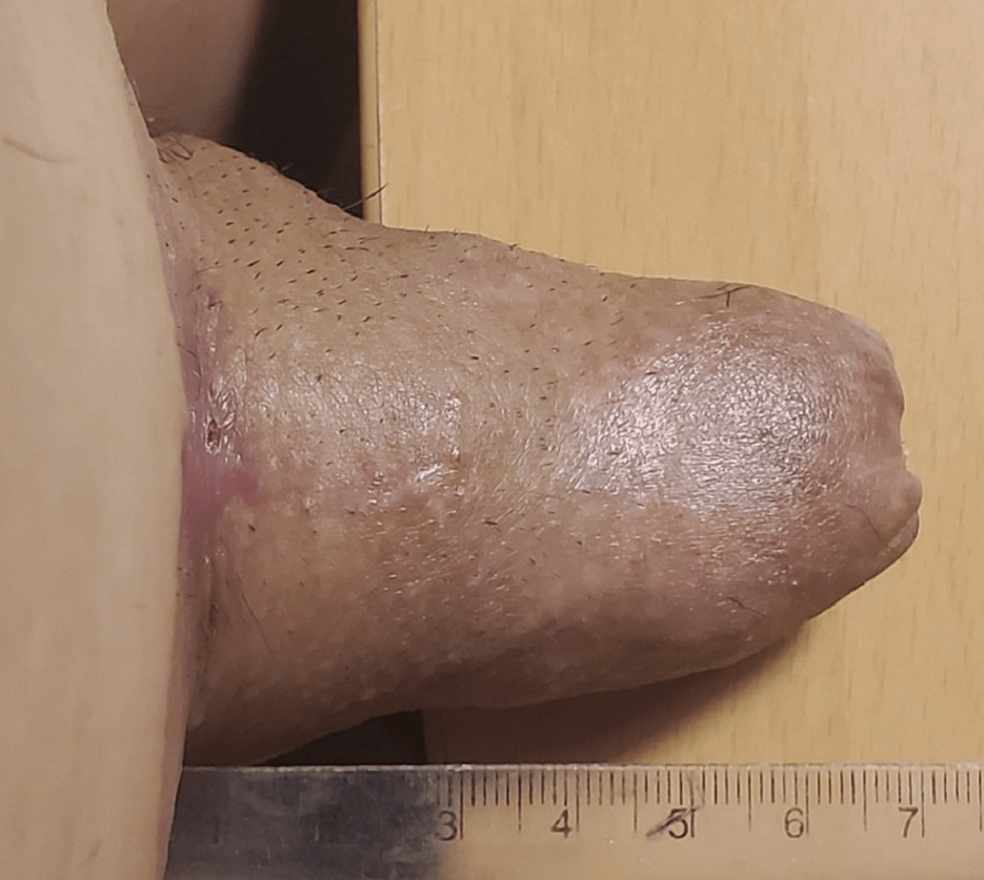
The penis after completely reconstructive procedures in the erection

One month after the first reconstruction, the patient was admitted to the Department of Urology for the second stage. We conducted the procedures the same as the treatment of the buried penis: Modified Lipszyc technique for the buried penis involved removing the thickened fascia penis and anchoring the deep face of the dermis to the proximal part of the fascia penis at the base of the penis (Figure 4). Pubic lipectomy involved removing the suspensory ligament of the penis and tacking the dermis to the underlying fascia (Figure 5). The outcomes of the two-stage reconstructive surgery were that the patient had a good urinary function and preserved erectile function (Figure 6). The length of the penis following the reconstruction is 5 cm in the flaccid state and 7 cm in the erect state (Figures 7-8).

## Discussion

The presence of microscopic polyangiitis is rarely associated with APS, since antiphospholipid antibodies were present in 17% of patients with primary systemic vasculitis. This condition affects small-sized vessels that almost invariably causes the damages of lung and kidneys, as well as cutaneous involvement [7]. CAPS, also known as Asherson’s syndrome, is a severe type of APS and rarely seen in clinical practice that affects <1% of patients with the diagnosis of APS [6]. Multiple thromboses coincide in microcirculatory fields, resulting in multiple organ failures fastly. The criteria for definite APS is at least one of the clinical criteria including the presence of vascular thrombosis or pregnancy morbidity, along with one of the laboratory criteria including the presence of antibodies. Antiphospholipid antibodies can not only be detected in APS but also can be found in healthy people, as well as in several clinical settings such as in individuals with a history of thrombosis and/or obstetrical complications or other autoimmune conditions (including systemic lupus erythematosus) [8]. Relying on the initial clinical and laboratory signs, we oriented the diagnosis to APS and confirmed the diagnosis based on repeated serum antibody tests at 12 weeks with anticardiolipin (+).

In this case, thrombotic events developed promptly in multiple blood vessels that manifested dry necrosis in the skin and combined with severe acute injuries of the lung and kidney. In addition, the immunoassay results also supported the diagnosis of CAPS, which had a mortality rate of up to 50%. This disease has been associated with several bacterial and viral infections such as the influenza A virus and Epstein-Barr virus [9]. In our patient, CAPS was likely induced by a viral infection of the upper respiratory tract. We conducted promptly a series of intensive treatments with a multi-modal approach: ventilatory support to improve respiratory function, continuous renal replacement therapy for kidney function, plasma exchange and immunosuppressive therapy to reduce adverse immune response, and heparin infusion for both the prevention and treatment of thrombotic events. For skin lesions, we also monitored and took care of the wound daily. These lesions were mainly superficial to the dermis, and most were treated conservatively without penile glandular necrosis. The ischaemic necrotic lesions of the penis due to the small vessels are uncommon, and particularly following APS is extremely rare in clinical practice. There was only one report of a case with total penile necrosis caused by CAPS [10].

Regarding the treatment for glandular necrosis, the treatment options have not yet been established. Several therapies have been described and utilized with positive outcomes. However, these therapies have only been reported in a small number of cases. The ultimate objective of them was revascularizing ischemic tissue by enhancing the arterial flow and venous drainage. Topical 10% testosterone undecanoate, intracavernous glycerol trinitrate, and several intravenous agents such as iloprost, pentoxifylline, and low-dose heparin, together with penile nerve block or hyperbaric oxygen, were reported to be conservative therapies for ameliorating ischemia of the glans [11]. In this patient, we could not preserve the glans penis. Reconstructive surgery was performed after the patient’s general condition was stable.

For treating penile defects, numerous surgical techniques were reported by utilizing different types of skin flaps such as local flaps or distant flaps. Penile reconstruction could be conducted with a split-thickness skin graft, full-thickness skin graft, scrotal flaps, and Cecil’s scrotal implantation [1]. We chose the two-stage surgery for this patient to reconstruct the penis after the glans loss. In the first procedure, we conducted the urethral reconstruction and also buried the penis into the healthy penile skin flap and scrotal flap. After the first stage, the patient urinated well and the penile skin was good enough for the next step. The second reconstruction was performed four weeks later. We utilized the procedures the same as the treatment of the buried penis for penis length augmentation [9]. The patient was quite satisfied after surgery in both erectile function and urinary function. This case is one of the first papers that report penile reconstruction to treat glandular necrosis as a manifestation of CAPS, an extremely rare condition.

## Conclusions

CAPS is a severe and quickly progressive type of APS, which leads to rapid death from multi-organ failure due to thrombosis. To achieve successful treatment, it is crucial to prompt diagnosis and requires the participation of many specialties. Penile glandular necrosis is also a rare complication of CAPS, needing timely treatment and appropriate reconstruction later when the patient’s general condition is stable.
